# Molecular and Environmental Mechanisms Regulating Puberty Initiation: An Integrated Approach

**DOI:** 10.3389/fendo.2019.00828

**Published:** 2019-12-06

**Authors:** Sarantis Livadas, George P. Chrousos

**Affiliations:** ^1^Endocrine Unit, Metropolitan Hospital, Athens, Greece; ^2^UNESCO Chair on Adolescent Health Care, University Research Institute of Maternal and Child Health and Precision Medicine, Aghia Sophia Children's Hospital, National and Kapodistrian University of Athens, Athens, Greece

**Keywords:** puberty, adrenarche, epigenetics, endocrine disruptor (ECD), stress

## Abstract

The mechanisms underlying the initiation of puberty, one of the cornerstones of human evolution, have not been fully elucidated as yet. However, recently, an accumulating body of evidence has helped unravel several critical aspects of the process. It is clear that a change in the pattern of pituitary gonadotropin secretion serves as a hormonal trigger for puberty induction. This change is directly guided by the hypothalamic GnRH pulse generation, a phenomenon regulated by the Kisspeptin-Neurokinin-Dynorphin (KNDy) system also in the hypothalamus. This represents the kisspeptin molecule, which is crucial in augmenting GnRH secretion at puberty, whose secretion is fine-tuned by the opposing signals neurokinin B and dynorphin. Recently, the novel kisspeptin inhibitory signal MKRN3 was described, whose role in puberty initiation provided further insight into the mechanistic aspects of pubertal onset. Furthermore, the description of higher inhibitory and stimulatory signals acting upstream of the KNDy neurons suggested that the trigger point of puberty is located upstream of the KNDy system and the GnRH pulse generator. However, the mechanism of pubertal onset should not be considered as an isolated closed loop system. On the contrary, it is influenced by such factors as adipose tissue, gastrointestinal function, adrenal androgen production, energy sensing, and physical and psychosocial stress. Also, fetal and early life stressful events, as well as exposure to endocrine disruptors, may play important roles in pubertal initiation, the latter primarily through epigenetic modifications. Here we present the available data in the field and attempt to provide an integrated view of this unique and crucial phenomenon.

## Introduction

According to Webster's dictionary, puberty is defined as “*the period of first becoming capable of reproducing, and is marked by maturation of the genital organs, development of secondary sex characteristics, acceleration in linear growth velocity, changes in affect, and, in the female, the occurrence of menarche*.” It is obvious that this period of quick and radical changes of human physiology, as well as of psychosocial and psycho-behavioral functions, is regulated by hormonal mechanisms. However, despite the major work carried out in this field during the past five decades and, notwithstanding the accumulation of a significant amount of data, there are still several gaps in our understanding of the mechanisms regulating this puzzling phenomenon.

At the level of physiology, puberty should not be considered a novel organismal process but rather a reawakening of a network developed already during fetal life. Indeed, in the developing brain the main components of the reproductive axis are apparently formed, including the hypothalamic Kisspeptin-Neurokinin-Dynorphin (KNDy) system and its downstream GnRH pulse generator in the arcuate nucleus of the hypothalamus. This pathway is active during infancy, a phenomenon that has been termed “mini-puberty,” but is turned off during the transition from infancy to the juvenile phase, as early as 4–6 months of life in the male and as late as 24 months in the female. This active inhibitory process is crucial for the sexually quiescent period of childhood, serving as a neurobiological brake of reproductive function. Its regression signals the onset of puberty (1). In our species, from an evolutionary point of view, childhood is essential for allowing the intellectual development and physical growth of an individual before attempting to reproduce (2). Accordingly, the mystery of puberty lies in the nature of the above neurobiological brake and its elimination at the end of the juvenile period.

Several factors, such as genetics, epigenetics and lifestyle, are implicated in the onset and completion of puberty, while stress and energy sensing processes also play important roles. We propose that during this crucial period of life there is a dynamic interplay of multiple factors whose elucidation will guide us to a more comprehensive recognition of the mechanisms regulating the onset and achievement of puberty.

## GnRH Pulse Generator

The function of the hypothalamic-pituitary-gonadal (HPG) axis is orchestrated by a diffuse network of hypothalamic neurons, which generates recurrent pulsatile discharges of the neuropeptide GnRH into the hypophysial portal circulation with a frequency of about 1 every 60–90 min. These secretory episodes 10 regulate the synthesis and secretion of pituitary gonadotropins into the systemic circulation, stimulating ovarian and testicular function. This key neuronal network is termed the “GnRH pulse generator” (3).

GnRH exerts its actions via its specific G-protein-coupled receptor (GnRH-R) located on the surface of the pituitary gonadotropin-secreting cells. GnRH is secreted into the hypophysial portal circulation, and its actions are reflected by the episodic systemic secretion of LH and FSH.

GnRH actions on pituitary gonadotrophs is a highly sophisticated system. Specifically, low GnRH levels induce dissociation of the Gas subunit of the GnRH receptor, intermediate concentrations induce dissociation of the Gaq subunit of the same receptor, while high concentrations induce dissociation of the Gai subunit (4). These different subunit dissociations modify GnRH release and regulate pulsatile gonadotropin secretion in a very “tight” manner (5). Indeed, one of the first stages of puberty is the observation of an increase in both observed frequency and amplitude of GnRH pulse generator bursts. These changes constitute an early neurobiological event in the initiation of puberty. It is evident that mutations of either the GnRH or its receptor gene may modify, *inter alia*, the timing of puberty (6). However, such genetic aberrations account for only a negligible proportion of subjects who experience either precocious or delayed puberty, strongly indicating that the GnRH pulse generator is influenced by upstream networks.

## Stimulatory Signals

### The KNDy System

Various hypothalamic factors influence GnRH secretion, while both excitatory and inhibitory trans-synaptic neuronal inputs modulate the function of the GnRH pulse generator. Among them, the immediate upstream and apparently most important system involved in the fine-tuning of GnRH secretion, is the KNDy system. The importance of Kisspeptin, neurokinin B (NKB) and dynorphin neuromodulators was conclusively confirmed when selective ablation of any of them in rats significantly decreased LH secretion. The discovery of kisspeptin secretion and its impact on the GnRH pulse generator was a major step in our understanding of the pubertal process (7, 8). Furthermore, the detection of NKB and dynorhin as reciprocal stimulatory (NKB) and inhibitory (dynorphin) signals fine-tuning kisspeptin oscillation, further elucidated KNDy function in the regulation of GnRH (9). The role of KNDY in humans is highlighted from the observations that loss-of-function mutations in the genes encoding for kisspeptin (KISS1), kisspeptin receptor (KISS1R), NKB (TAC3), or NKB receptor (TACR3) are associated with delayed puberty and hypogonadism (10). Elegant experiments carried out in mice have shown that while kisspeptin neurons in mice inherently exhibit intracellular calcium oscillations at 8-min intervals, the GnRH/LH pulse frequency in the same animal model was about every 20 min, suggesting that kisspeptin, although essential for GnRH release, it is not the only factor directly contributing to GnRH oscillations (11).

The quiescence of the gonadotropin axis during childhood, is followed by a reawakening of the GnRH pulse generator at the onset of puberty, owing to enhanced stimulatory influences on the GnRH neuronal network. One of the cardinal stimulants is glutamate, which is the main excitatory neurotransmitter in the brain. Interestingly, hypothalamic release of glutamate is greatly increased during puberty, as administration of N-methyl-D-aspartate (NMDA), a glutamate receptor agonist, in juvenile monkeys, led to premature puberty initiation (12).

Transcription factors are also impacted in pubertal onset. Thus, a significant increase in the expression of two such factors in the mediobasal hypothalamus is noted during puberty. Namely, interferon regulatory factor 2 binding protein-like (EAP1) is produced in kisspeptin neurons, which, when silenced through knock-out experiments, brings about menstrual irregularities in female monkeys (13). The role of thyroid transcription factor-1 (TTF-1) was delineated when similar knock-out experiments of this factor from hypothalamic neurons showed decreased Kiss1 expression and delayed puberty in the affected animals (14).

A role for glial cells in puberty has also been proposed, as a tight association has been observed between these cells and GnRH neurons. Also, it has been shown that both astrocytes and ependymoglial cells lining the ventrolateral surface of the third ventricle, augment GnRH secretion via adhesive cell-cell interactions by releasing ATP, prostaglandins, and growth factors, as well as cell and synaptic adhesion molecules (15).

## Inhibitory Signals

The identification of a neurobiological brake restraining the onset of puberty points to the presence of inhibitory signals down-regulating KNDy-GnRH secretion. Among them, macorin-3 (MKRN3) plays an important role, as its expression in the hypothalamic arcuate nucleus decreases abruptly before the commencement of puberty, followed by a stable decline of circulating MKRN3 levels during the progression of puberty (16). MKRN3 encodes a protein involved in ubiquitination and cell signaling. Its gene is parentally imprinted, i.e., maternal allele is methylated and silenced, while the paternal allele is expressed. Loss of function mutations of MKRN3 have been associated with precocious or early puberty and represent the most common (up to 46%) familial etiology of central precocious puberty (17). Moreover, its central role in the natural course of puberty is emphasized by the observation made through a large genome-wide analysis study (GWAS), that single-nucleotide polymorphisms (SNPs) of the MKRN3 region affect the age of menarche in healthy girls (18).

One of the major inhibitory neurotransmitters regulating brain function is GABA. GABAergic neurons are linked to GnRH pulse generator oscillation, as GABA receptors are expressed on GnRH neurons (19). Furthermore, the administration of bicuculline, a GABA receptor blocker, radically increased kisspeptin production, implicating GABA in the onset of puberty. Additionally, in rhesus monkeys, the hypothalamic tone of GABA is up-regulated during juvenile development, while its inhibition leads to precocious puberty (20). In humans, evidence was recently provided from a GWAS study, in which it was observed that SNPs of the GABA signaling pathway were associated with age at menarche (18).

Studies in birds revealed another component of the neurobiological “brake,” namely, the gonadotropin-inhibitory hormone (GnIH) and the hypothalamic RF amide-related peptides (RFRP), which are considered the homolog genes of mammalian species. Indeed, RFRP hypothalamic neurons discharge RFRP1 and RFRP3 peptides, which exert a negative effect on GnRH secretion (21). Finally, a role for opiatergic neurons has also been unraveled, as these neurons block GnRH activity either directly or indirectly, the latter by suppressing kisspeptin release through a paracrine/autocrine mode of action (22).

## Genetics

Obviously, gene mutations affecting components of the above-described factors will lead to disruptions of the pubertal process. Thus, rare mutations of more than 20 genes that participate in the intact function of the HPG axis have been reported and used as natural prototypes for the elucidation of mechanisms regulating puberty, as well as the overall function of the axis (23). Interestingly, mutations of genes that interrupt embryonic migration of GnRH-producing cells from the nasal placode to the hypothalamus have shed light on the underlying causes of Kallman syndrome, while mutations of growth factors responsible for pituitary development (e.g., PROP1, HESX1, and SOX2) have elucidated the molecular machinery of multiple pituitary hormone deficiencies (24). Furthermore, mutations of the genes discussed above, including kisspeptin (KISS1), neurokinin B (TAC3), and GnRH (GNRH1), along with those of their receptors (KISSR, TACR3, and GNRHR), have underlined the role of these factors in pubertal onset (25).

Twin studies have shown that pubertal onset is highly correlated between related individuals, with genes being significantly implicated in this variation (explaining up to 60–80% of the variance) (26). However, the above-described mutations, originating from the study of very rare clinical disorders, have revealed only a small proportion of the genes involved in this process. On the other hand, the advent of GWAS, in which millions of SNPs across the genome are simultaneously analyzed, provided some interesting data. By using timing of menarche as a biomarker of puberty, it has been found that the latter is a highly polygenic childhood trait, involving more than 300 independent signals in a large GWAS of 370,000 women. Of note, some of these signals were associated with genes of the KNDy-GnRH system, however certain other regions, though identified, play as yet unknown roles (27).

Within the same context, we should not neglect the impact of gender on puberty. In nature, earlier puberty is usually observed in females rather than males, while in humans, pubertal onset usually occurs 1–2 years earlier in girls than in boys, with girls reaching reproductive maturity earlier than boys (28). Of note, girls are more prone to develop precocious puberty, whereas boys are more predisposed to delayed puberty. It has been postulated that the neurobiological “brake” producing human childhood, is looser in females than in males; interestingly, gonadotropin levels are significantly higher in agonadal girls compared to agonadal boys (29). This sex difference in the strength of the neurobiological “brake” on prepubertal GnRH release has been attributed to the greater exposure of the fetal male hypothalamus to testosterone concentrations.

## Extrinsic Factors

To sum up, pubertal onset is characterized by an abrupt decrease of inhibitory trans-synaptic inputs, followed by a boost of stimulatory inputs to the GnRH neuronal network. However, cues from the periphery that may lead to changes in the activity of these inherent systems have not yet been discussed. By definition, an intact GnRH network and the proper functioning of its molecular machinery, including neurotransmitters, neuromodulators, and paracrine interactions, constitute a *sine qua non* for the evolution of the process of puberty during our speciation (30). Granted, however, that 20–40% of the variation of pubertal onset is not genetically-dependent (31), it is obvious that there should be other key players that actively participate in its regulation. Data obtained from elegant studies have shown that several extrinsic factors participate in the orchestration of the pubertal process.

## Epigenetics

Epigenetics are involved in almost every function of the developing organism including puberty. The term epigenetics refers to any modification of gene expression, which cannot be attributed to changes of DNA sequence itself. Epigenetic modifications include DNA methylation, chromatin packaging, non-coding RNAs, non-coding DNA regulatory sequences, and long-range transcriptional regulators (enhancers and insulators) (32). The existence of the childhood neurobiological “brake” that represses the onset of puberty is well-documented. Since DNA hypermethylation of gene promoters is associated with gene silencing, a role for epigenetics in menarche was studied and partially elucidated. In fact, the methylome profiling of girls revealed a widespread pattern of DNA hypermethylation, as well as methylation of several “zinc finger”-containing genes. Furthermore, age of menarche was inversely associated with DNA methylation, whereas methylated phosphate-guanine (CpG) dinucleotide groups were associated with changes in gonadotropin and testosterone levels in boys entering puberty (33).

Experiments in monkeys have shown that methylation and histone modification of the GnRH gene itself affects its function (34). In accordance with these findings, administration of chemicals that modify epigenetic marks in rodents resulted in earlier onset of puberty, whereas a global inhibition of DNA methylation in prepubertal rats had the opposite results (35). The main epigenetic driver of these actions seems to be a group of transcriptional suppressor protein complex known as the “polycomb” group. More precisely, in rats, the onset of puberty is triggered by DNA methylation and decreased hypothalamic expression of two key polycomb group genes, Eed and Cbx7, which, in turn, activate lysine modifications of histone H3, whereupon the Kiss1 gene begins to express kisspeptin mRNA (36, 37). It must be borne in mind that the epigenome is constantly reprogrammed throughout life and that its plasticity may serve as a protective mechanism to allow pubertal onset take place at the most appropriate time for the developing organism.

## Endocrine Disruptors

In the modern era everyday life, humans are increasingly exposed to numerous chemicals (over 1,000) through ingestion, inhalation, and dermal contact. A significant number of these compounds have the ability to disrupt the endocrine system, including some of its most important functions. The so-called endocrine disrupting chemicals (EDCs), contaminate many regions of our planet and interfere with every aspect of hormone action. EDCs can bind to hormone receptors leading to either activation or suppressions of the natural hormones or may modify their degradation, thereby leading to alterations of normal hormonal signaling. Among them, pesticides [dichlorodiphenyl trichloroethane (DDT) and its primary metabolite DDE], polybrominated flame retardants (polybrominated biphenyls PBB and PBDE, dioxins, phthalate esters, and plasticizers (phthalates, bisphenol A) have been studied specifically in relation to gonadal differentiation and puberty, given that they may exert estrogenic, androgenic, anti-androgenic, and other actions (38).

The available results regarding the EDCs are complex and cannot unequivocally prove causality for distinct reasons. First, EDCs exert their actions in non-monotonic dose-response curves, as hormones interact with and activate their receptors in a non-linear fashion. For example, in rats, neonatal exposure to BPA for 2 weeks led to delayed puberty at a low dose, but earlier puberty when a higher dose was administered. Similarly, lower doses of phthalates were associated with early puberty, whereas the opposite effect was observed with higher doses (39). Second, timing of exposure has a substantial effect on the final outcome. Thus, exposure to high PBDE concentrations *in utero* and/or through breastfeeding led to premature adrenarche, and/or earlier menarche by 1 year compared to unexposed girls. Of interest, menarche was earlier in those exposed only *in utero* compared to controls, but later in those exposed both pre- and postnatally, suggesting that timing and duration of exposure has a significant impact on the developing human (40).

Finally, in humans, it is difficult to provide evidence of a causal relation, granted that subjects are exposed to many chemicals at the same time. Moreover, mostly cross-sectional rather than prospective studies have been carried out assessing potential cofounders. For example, with regard to BPA, higher serum or urinary BPA levels were found in girls with precocious puberty, suggesting a possible role of BPA in this event. This finding was supported by findings from the NHANES study, in which higher urinary BPA levels were associated with earlier menarche. By contrast, a study from China showed delayed menarche in girls with increased BPA levels, while in certain other studies, no association between BPA levels and puberty changes has been disclosed (41).

Although contemporary children are developing signs of puberty onset earlier than in past decades, a trend toward later ages of completion of puberty has also been observed (42, 43). The above-described age distribution toward younger ages for pubertal onset stages together with an age distribution of a later completion of puberty has been partially attributed to exposure to EDCs. It was hypothesized that exposure to negative environmental cues in the early years of life might translate to an earlier puberty and reproductive maturity, because of “anticipating” environmental danger. On the other hand, the same early signs at a period closer to or during puberty, this phenomenon might be construed as a higher risk for a difficult pregnancy and/or a sick child, leading to a later puberty completion (44). Because humans are exposed to one or more EDCs at different levels and time periods, an apparent role of EDCs in the above changes could certainly be postulated (45).

## Adipose Tissue

The existence of a direct relation between pre-adolescent weight gain and the age of pubertal onset was noted several decades ago. The increasing global childhood obesity epidemic has been implicated as a cause of a lower age of pubertal onset among the contemporary population. Data originating from prospective studies demonstrated that an excessive increase in BMI during the childhood years led to earlier puberty development (46). Furthermore, data from GWAS have shown that the BMI-increasing allele of several genetic loci was associated with earlier age at menarche (47). Indeed, according to the “somatometer hypothesis,” a certain amount of fat mass is critical for initiation of puberty (48). The key player in this process is leptin, which is secreted by adipocytes and acts as a messenger to the hypothalamus transmitting information on fat mass and energy status.

Circulating leptin concentrations increase gradually with age prior to puberty, and this is suggestive of the presence of a leptin concentration threshold acting as a pubertal onset trigger (49). Conversely, patients carrying loss-of-function mutations of leptin or its receptor do not develop puberty, while exogenous administration of this hormone to patients with leptin deficiency promoted pubertal development. This phenomenon was attributed to a direct action of leptin on neuronal nitric oxide synthase neurons of the hypothalamic preoptic region, leading to increased LH secretion, probably independently of the KNDy system. The action of leptin on pubertal onset is essential but permissive rather than regulatory, granted that in patients with lipodystrophies, where minimal amounts of leptin are produced, pubertal development is normal (50).

Another factor related to adipose tissue is DLK1, or preadipocyte factor 1 (Pref-1), which is a transmembrane protein encoded by an imprinted, paternally expressed gene: it is located on the long arm of chromosome 14 (14q32.2) and acts as an adipogenesis gatekeeper by preventing adipocyte differentiation; this factor is also expressed in kisspeptin cell lines and several hypothalamic nuclei (51). Of note, in GWAS, an association of this gene with puberty was demonstrated, while, recently, 10 adult women with a history of precocious puberty and DLK1 mutations were reported. Interestingly, these women, who suffered from obesity, insulin resistance, dyslipidemia and type 2 diabetes mellitus, predictably had an adverse metabolic profile. These findings were similar to those observed in Dlk1-null mice, which developed insulin resistance, glucose intolerance, and increased circulating levels of triglycerides, cholesterol, and free fatty acids in adulthood, suggesting that DLK1 may represent a long sought link between reproduction and metabolism (52). Undoubtedly, more studies are needed to delineate this association.

## The Gastrointestinal Axis

A major discovery of the last decade is the emergence of the gut microbiome as a major factor influencing homeostasis. Indeed, this diverse ecosystem hosted in the human body interacts with the brain and, hence, the term “microbiome-gut-brain axis.” Indeed, there is a multifaceted crosstalk between the gut microbiome and the brain and a role of microbiota in puberty is more than likely. During puberty two profound changes of the microbiome are observed, first, we have shifts from aerobic and facultative anaerobic to anaerobic species, and second, a greater abundance of *Bifidobacterium* and *Clostridium* in adolescents than in adults. Furthermore, a gender-specific gut microbiome begins to develop at this time. Of note, the transplant of gut microbiota from adult male rats to immature female animals led to increased testosterone levels, similar to those observed in males, implying a role of microbiota in hormonal production (53). More surprisingly, the administration of specific microbial species has been shown to modify brain function by modulating the levels and availability of neurotransmitters critical in the process of puberty, such as glutamate and GABA (54). A role of the microbiome in pubertal development cannot, thus, be excluded, although more studies are needed to clarify this relationship.

Another key regulator of the gastrointestinal axis and pubertal development is ghrelin, a peptide secreted predominantly by the stomach, which acts mainly as a signal of energy deficiency, with its concentrations increasing during fasting, and falling post food ingestion. It appears that ghrelin and leptin act as reciprocal regulators of energy homeostasis by exerting opposing effects. With regard to puberty, the ghrelin receptor (GHSR-1a) is expressed in all components of the hypothalamic-pituitary gonadal axis, while ghrelin exerts a negative effect on LH pulsatility and sex steroid production (55, 56).

From the same point of view, an emerging role for fibroblast growth factor 21 (FGF21) in puberty initiation has been recently demonstrated. FGF21 is a fasting-induced hepatokine, which regulates a systemic response to fasting by increasing ketogenesis, suppressing growth, and promoting torpor (57). Regarding puberty, it has been shown that FGF21 acts on the suprachiasmatic nucleus (SCN) in the hypothalamus by suppressing kisspeptin signaling cascade, whereas animals lacking the FGF21 co-receptor, β-Klotho, in the SCN are refractory to the inhibitory effect of FGF21 on female fertility (58). Thus, FGF21 seems to actively participate in the gastrointestinal-neuroendocrine axis that modulates puberty and reproduction in both sexes.

## Adrenarche

Adrenarche denotes the activation of the *zona reticularis (ZR)* of the adrenal gland, which results in a gradual, age dependent increase of adrenal androgen production: it represents a recent evolutionary development since it is observed only in higher primates. Adrenarche is clinically manifested by pubic hair development, which is called “pubarche.” Although these two terms are frequently used as synonysms, they represent distinct entities; they, thus, should not be confused (59).

Sixty years ago, F. Albright observed the development of pubic hair in girls with gonadal dysgenesis under the influence of adrenal androgens (DHEA and DHEAS) in the absence of gonadal hormones. As a result, he drew a sharp distinction between the pubertal process of “gonadarche,” meaning gonadal activation and, consequently, the ability to reproduce, and adrenarche. The driving force of adrenarche has not as yet been elucidated, although several factors, such as prolactin, estrogens, epidermal growth factor, angiotensin, gonadotropins, pro-opiomelanocortin (POMC)-related peptides, growth hormone (GH), and insulin growth factor 1 (IGF1), and insulin, have been linked to ZR development and function. Nevertheless, a distinct permissive role of both the CRH/ACTH axis and adipose tissue has been documented, although these do not constitute the main regulators of this phenomenon (60).

Of great interest is the recent discovery of the kisspeptin signaling system in the adrenals during fetal life. Kisspeptin expression leads to increased DHEAS production from human fetal adrenal cells and seems to be a regulator of the feto-placental unit during pregnancy (61). Whether, there is a role for kissppetin in adrenarche, which, in fact, represents the regeneration of adrenal androgen production machinery established in the fetal adrenal, has not been verified to date.

The fact that serum levels of adrenal androgens start to rise approximately 2 years prior to gonadarche suggests a possible link between these two biologic processes. However, adrenarche does not constitute a prerequisite for gonadarche, as gonadarche and physiologic puberty proceeds normally in clinical entities in which adrenarche is absent. Furthermore, the age of gonadarche and menarche in children with premature adrenarche or pubarche was not different from that in the general population (62). However, in girls who expressed both premature adrenarche and pubarche, earlier menarche was noted, implying a role of adrenal androgen secretion in the initiation of gonadal action. Moreover, in girls with PP born small for gestational age (SGA) had significantly advanced age of menarche in comparison to PP girls born with a normal birth weight, suggesting a link between prenatal growth restriction, premature activation of the ZR, and earlier gonadal maturation (63).

## Fetal Life/Psychosocial Stress

A significant amount of evidence shows that prenatal and early-life adversity is associated with early reproductive maturation. Based on the concept of the developmental origins of health and disease (DOHaD), it has been suggested that the developing fetus modifies homeostatic system activities in response to early life cues. These stressful events associated with intrauterine growth restriction, result in fetal programming of endocrine axes for a stressful extrauterine life, functional changes that are associated with an increased risk of chronic mental and physical non-communicable diseases (64). The precocity in sexual maturation observed in such children can be explained by the following concept: as fetal growth restriction is associated with increased risk of disease and mortality, the developing subject should become capable of reproduction at an earlier stage than anticipated in order to complete the life cycle (65). Indeed, experiments in rats have shown that maternal undernutrition modified GnRH transcript levels and normal neonatal leptin surge, with an impact on neuronal regulation of both metabolism and reproduction during the early stages of life (66).

On the other hand, as many phenomena occurring in nature exhibit *U*-shaped dose-response curves, excessive gestational weight gain during pregnancy is also an independent risk factor for early menarche. Furthermore, exposure to maternal obesity and/or hyperglycemia increases the risk for premature adrenarche. In addition, experiments in rats showed that animals whose mothers were fed a high-fat diet during pregnancy developed puberty earlier than their control peers (67). Accordingly, certain environments during pregnancy have been shown to perturb endocrine homeostasis, while there are clear interactions between the prenatal nutritional environment and post-natal endocrine and reproductive function (68).

The study of girls born with low birth weight have shown that they exhibit a higher risk of developing premature adrenarche, earlier menarche, insulin resistance, hyperandrogenemia, and an unfavorable metabolic profile (69). The role of insulin in this setting has been elucidated by experiments carried out in this subset of prepubertal girls, where the administration of the insulin sensitizer metformin resulted in a significant postponement of breast development, followed by a mean delay of menarche timing by 1 year (70). Interestingly, these findings were associated with decreased circulating leptin and insulin-like growth factor-I levels. Also of interest, the administration of metformin led to significant improvements in BMI, waist circumference, circulating androgens, and plasma lipid profile, which lasted up to 4 years of follow-up (71). It, thus, seems that the disruption of adipose tissue function due to early life stressful events, causing insulin resistance and decreased leptin secretion, affects metabolic, and reproductive function during adolescence.

Another aspect of the combination of stressful events and the post-natal environment has been observed in young girls who migrated from a poor to a developed country, a change associated with a 10–20-fold increased risk of precocious puberty (72). This finding is in agreement with data coming from prospective studies and demonstrating that girls who were born with low birth weight but who became overweight prepubertally entered puberty earlier than their peers with low birth weight who remained lean (73). It appears that exposure to prenatal and/or early-life deprivation and nutritional excess in childhood disrupts maturation of reproductive axis.

Finally, the impact of social stress on pubertal onset should not be overlooked, as life stressors affect brain structure and function. Thus, subjects of a low socioeconomic status or those who had been exposed to at least two traumatic stressful events developed earlier puberty (74, 75). Therefore, it has been hypothesized that stressful events advance sexual maturation (stress acceleration hypothesis), probably as an evolutionary adaptive response (76).

## Energy Sensing

The ability to reproduce constitutes the final step of pubertal development. Because reproductive capacity is a highly energy-demanding process, conditions of negative energy balance, such as eating disorders, or high-intensity exercise may lead to reproductive suppression and resultant hypofertility/hypofecundity. Such extrinsic stressful events suggest that anticipations of distorted energy sensing, and distorted fuel-sensing pathways are reasonable candidates for both detection of environmental nutrition deprivation stress and as mediators of adaptive responses (77).

At the cellular level, several key components regulate energy and nutrient sensing and the equilibrium between anabolic and catabolic signals. These include the mammalian target of rapamycin (mTOR) pathway, which is the main cellular nutrient sensor (which senses availability of glucose and amino acids) and controls protein and lipid biosynthesis, and the AMP kinase (AMPK) pathway, which is the main energy sensor (which senses AMP vs. ATP levels). The latter is stimulated by oxidative stress and controls mTOR The mTOR sensor pathway can be activated by glucose, amino acids, and hormonal signals (IGF-1, insulin) and can influence downstream effects on protein translation and cell growth (78). The available evidence suggests that suboptimal activity of components of the above pathways can cause failure to recognize abnormal nutrient/fuel levels, leading to maladaptive cellular responses, such as insulin resistance (79).

Since the nutrient and energy sensing systems are crucial for cellular integrity and function, we have speculated whether they could participate in the process of puberty. Indeed, the mTOR/AMPK pathways are partially regulated by Sirtuin 1 (Sirt1), which is an NAD-dependent class III deacetylase that also functions as a cellular energy sensor activated during fasting or caloric restriction to increase fatty acid oxidation and gluconeogenesis and suppress insulin secretion, insulin action, and adipogenesis (80). Apart from its ubiquitous expression in adipose tissue, liver, and muscle, Sirt1 is also expressed in the hypothalamus. Specifically, its activity increases in the hypothalamus during fasting, while deletion of Sirt1 in mice in pro-opiomelanocortin (POMC) hypothalamic neurons decreases energy expenditure and increases susceptibility to diet-induced obesity, whereas its deletion in Agouti-related peptide (AgRP) neurons has the opposite effect (81). The latter was anticipated, given the contrasting roles of these two neuronal populations in anabolic and catabolic circumstances. Furthermore, SIRT1 is expressed in hypothalamic Kiss1 neurons and interact with the Polycomb silencing complex to decrease Kiss1 promoter activity. It has been found that early-onset overnutrition enhances Kiss1 expression and advances puberty, whereas undernutrition raises SIRT1 levels, protracts Kiss1 repression and delays puberty (82).

Regarding astrocytes, which, as stated above, exert an inhibitory role in GnRH secretion, experiments in animals have shown that decreased expression of Sirt1 led to reduced hypothalamic Kiss1 levels and suppressed reproductive function. Interestingly, these changes were accompanied by significant alterations of carbohydrate metabolism, implying a combined role of nutrient/energy sensing in both metabolism and reproduction (83). More experiments are needed to clarify the role of the elegant molecular machinery of nutrient/energy sensing in the pubertal process.

## Synthesis of Data

**Figure 1:**
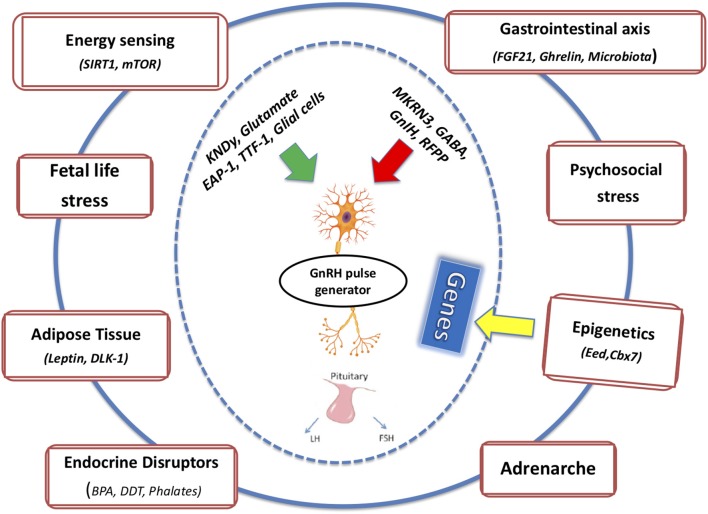
The integrated effects of multiple intrinsic and extrinsic factors on mechanisms of pubertal onset and completion are depicted.

In the following section, we will attempt to connect the pieces of the puzzle described above and the potential crosstalk among these factors on the basis of the pubertal process (Figure 1). Regarding endocrine disruptors, given the significant amount of evidence demonstrating a direct relation between exposure to EDCs and obesity, the term metabolism-disrupting chemicals (MDCs) has been introduced to underscore the ability of numerous endocrine disruptors to promote obesity and an adverse metabolic profile. EDCs exhibit antiandrogenic and xenoestrogenic/anti-estrogenic actions: because sex steroids directly influence adipose tissue function, an effect of EDCs was expected (84).

Several studies carried out in both humans and animals have shown that EDCs restrain the androgen receptor pathway, augment, or block the estrogen pathway, and/or decrease androgen levels through the up-regulation of the aromatase enzyme. These hormonal modifications lead to an increased number and size of adipose cells, altered adipocytokine production, reduction of basal metabolic rate, and modification of appetite and satiety signaling (85). Although the available data derived from animal studies are more solid than those obtained from human epidemiological studies, a link between EDCs and an adverse metabolic profile is predicted.

A striking link has been discovered in animal models between energy sensing and exposure to EDCs. Exposure of mice to BPA during the prenatal period modified hypothalamic orexigenic and anorexigenic neuron structure and function, leading to distorted food intake and increased leptin and insulin levels during puberty. In concert with these findings, exposure of adult mice to TBT induced alterations of the leptin-NPY pathway and caused disturbances of the hypothalamic-pituitary-adrenal axis (86).

Another class of EDCs that gained wide public attention over the last decade is composed of advanced glycated end products (AGEs), which are products of non-enzymatic glycation and oxidation (glycoxidation) of proteins and lipids and are ingested in the circulation from everyday food consumption, especially from sweet and savory snacks and heat-processed foods (87). AGEs exhibit strong oxidative, proinflammatory, and proatherogenic properties and amplify insulin resistance. Interestingly, recent data reveal an emerging role in autophagy through the mTOR/AMPK pathways, connecting this class of EDCs with the nutrient and energy-sensing cellular machinery (88).

Dietary exposure of the gastrointestinal tract to EDCs modifies the composition of microbiota, and these changes have been coupled with abnormalities in cytokine production and unfavorable hepatic lipid and carbohydrate metabolism. Finally, recent evidence indicates that exposure to EDCs during development can not only directly harm the exposed individual, but also the individual's offspring and future generations, an action brought about through epigenetic mechanisms (89).

Reproductive maturation is influenced by early-life events, while fetal life stress is usually expressed as low birth weight, as a result of intrauterine growth restriction. Human and experimental evidence shows that prenatal early-life adversity is associated with earlier reproductive maturation. In fact, in girls born SGA, premature adrenarche and earlier menarche were observed. Likewise, unfavorable fetal programming takes place in excessive weight gain during pregnancy. It has been reported that offspring of women with obesity and/or hyperglycemia during pregnancy have a higher risk of developing earlier pubarche compared to controls. Furthermore, excessive gestational weight gain during pregnancy was an independent risk factor for early menarche (90–93).

While birth weight, preadolescent weight, and puberty are sequentially interrelated, there are clear interactions between the prenatal and post-natal nutritional environments. In SGA girls who were overweight prepubertally, pubertal onset began almost 1 year earlier than in their SGA peers with normal BMI before adolescence. Analogous findings were obtained in girls exposed to maternal obesity and high weight gain during pregnancy. Those who were overweight prepubertally developed earlier menarche compared to those with a normal BMI (94).

The association between adverse fetal programming and impaired nutrient and energy sensing and pubertal outcome has been demonstrated in a cohort of SGA girls in whom the administration of metformin, a potent insulin sensitizer, resulted in a one-year delay of menarche, associated with decreased circulating leptin and IGF-1 levels. Of interest, these intriguing results lasted for 4 years, as treated girls had 50% less fat, lower IGF-I, and androgen concentrations and exhibited a favorable metabolic profile (95). Regarding the connection of the nutrient/energy-sensing system with stress at the molecular level, a central role of SIRT1 has been discovered. SIRT1 deacetylates the glucocorticoid receptor and hence enhances its activity; the latter is acetylated in the early morning hours by the main central circadian system transcription factor CLOCK-BMAL complex. Accordingly, an important link between energy sensing, circadian rhythms, and glucocorticoids, the main mediators of stress response, has been revealed (96, 97).

A critical role of all the above-described phenomena are attributed to epigenetic mechanisms. In a recent GWAS, three imprinted gene regions, namely, MKRN3-MAGEL2, DLK1, and KCNK9, were associated with age at menarche in women. In fact, only the paternally-inherited alleles of MKRN3-MAGEL2 and DLK1, along with the maternally-expressed KCNK9 allele, were related to the timing of menarche, clearly showing the impact of epigenetics on puberty. Furthermore, it has been demonstrated that certain intrauterine environments during pregnancy perturb epigenetic remodeling in the embryo, having lasting impacts on both male and female offspring physiologies. In addition, the epigenetic response to maternal care does not affect a limited number of candidate gene promoters, but rather modifies broad genomic areas including transcriptional and intragenic sequences, including areas distant from transcription start sites (98). Furthermore, parental lifestyle factors, notably maternal and paternal nutrition, have been shown to alter the epigenome.

Genes with important metabolic actions have been shown to be susceptible to modification by epigenetic mechanisms during the juvenile and pubertal periods. For example, folic acid supplementation administered to juvenile offspring can increase PPARα and GR promoter methylation in the liver and decrease insulin receptor promoter methylation in the liver and fat (99). Progressive epigenetic changes involving DNA methylation, histone modifications, chromatin remodeling, and transcriptional changes have been described in rats with intrauterine growth restriction, during development of type 2 diabetes mellitus (100).

## Conclusions

Puberty, which constitutes a major milestone of human growth and development, has been directly linked to specific health consequences later, in adulthood (101). We support the notion that exposure of a susceptible genetic background to unbalanced environmental factors, such as fetal stress, psychosocial stress, and EDCs, may distort nutrient/energy sensing, gastrointestinal function, adrenal androgen production and other functions and, through them, produce pathologic manifestations. It is the sum total of multiple signals, that through “adaptive” epigenetic adjustments may positively, or negatively modify pubertal onset and completion. Although the mechanisms underpinning this array of associations are not fully understood, puberty should not be considered a simple lifting of an inhibitory process. Rather, a more integrated multidimensional approach involving the interaction of multiple intrinsic and extrinsic factors with the pubertal machinery, is required to help us understand and confront the various medical and psychological challenges associated with precocious or delayed puberty.

## Author Contributions

All authors listed have made a substantial, direct and intellectual contribution to the work, and approved it for publication.

### Conflict of Interest

The authors declare that the research was conducted in the absence of any commercial or financial relationships that could be construed as a potential conflict of interest.

## References

[1] Livadas S Chrousos GP. Control of the onset of puberty. Curr Opin Pediatr. (2016) 28:551–8. 10.1097/MOP.0000000000000386 27386974

[2] Worthman CM Dockray S Marceau K. Puberty and the evolution of developmental science. J Res Adolesc. (2019) 29:9–31. 10.1111/jora.12411 30869841 PMC6961839

[3] Maggi R Cariboni AM Marelli MM Moretti RM Andrè V Marzagalli M. GnRH and GnRH receptors in the pathophysiology of the human female reproductive system. Hum Reprod Update. (2016) 22:358–81. 10.1093/humupd/dmv059 26715597

[4] Herbison AE. The gonadotropin-releasing hormone pulse generator. Endocrinology. (2018) 159:3723–36. 10.1210/en.2018-00653 30272161

[5] Kim HG Bhagavath B Layman LC. Clinical manifestations of impaired GnRH neuron development and function. Neurosignals. (2008) 16:165–82. 10.1159/000111561 18253056

[6] Silveira LF Trarbach EB Latronico AC. Genetics basis for GnRH-dependent pubertal disorders in humans. Mol Cell Endocrinol. (2010) 324:30–8. 10.1016/j.mce.2010.02.023 20188792

[7] de Roux N Genin E Carel JC Matsuda F Chaussain JL Milgrom E. Hypogonadotropic hypogonadism due to loss of function of the KiSS1-derived peptide receptor GPR54. Proc Natl Acad Sci USA. (2003) 100:10972–6. 10.1073/pnas.1834399100 12944565 PMC196911

[8] Seminara SB Messager S Chatzidaki EE Thresher RR Acierno JS Jr Shagoury JK. The GPR54 gene as a regulator of puberty. N Engl J Med. (2003) 349:1614–27. 10.1056/NEJMoa035322 14573733

[9] Navarro VM. Interactions between kisspeptins and neurokinin B. Adv Exp Med Biol. (2013) 784:325–47. 10.1007/978-1-4614-6199-9_15 23550013 PMC3858905

[10] Ruka KA Burger LL Moenter SM. Regulation of arcuate neurons coexpressing kisspeptin, neurokinin B, and dynorphin by modulators of neurokinin 3 and κ-opioid receptors in adult male mice. Endocrinology. (2013) 154:2761–71. 10.1210/en.2013-1268 23744642 PMC3713217

[11] Han SK Lee K Bhattarai JP Herbison AE. Gonadotrophin-releasing hormone (GnRH) exerts stimulatory effects on GnRH neurons in intact adult male and female mice. J Neuroendocrinol. (2010) 22:188–95. 10.1111/j.1365-2826.2009.01950.x 20041983

[12] Claypool LE Kasuya E Saitoh Y Marzban F Terasawa E. N-methyl D,L-aspartate induces the release of luteinizing hormone-releasing hormone in the prepubertal and pubertal female rhesus monkey as measured by *in vivo* push-pull perfusion in the stalk-median eminence. Endocrinology. (2000) 141:219–28. 10.1210/endo.141.1.7231 10614642

[13] Dissen G Lomniczi A Heger S Neff T Ojeda S Hypothalamic EAP1 (Enhanced at puberty 1) is required for menstrual cyclicity in non-human primates. Endocrinology. (2011) 153:350–61. 10.1210/en.2011-1541 22128022 PMC3249687

[14] Mueller JK Dietzel A Lomniczi A Loche A Tefs K Kiess W. Transcriptional regulation of the human KiSS1 gene. Mol Cell Endocrinol. (2011) 342:8–19. 10.1016/j.mce.2011.04.025 21672609 PMC3148268

[15] Ojeda SR Lomniczi A Sandau U. Contribution of glial-neuronal interactions to the neuroendocrine control of female puberty. Eur J Neurosci. (2010) 32:2003–10. 10.1111/j.1460-9568.2010.07515.x 21143655 PMC3058235

[16] Abreu AP Macedo DB Brito VN Kaiser UB Latronico AC2. A new pathway in the control of the initiation of puberty: the MKRN3 gene. J Mol Endocrinol. (2015) 54:R131–9. 10.1530/JME-14-0315 25957321 PMC4573396

[17] Macedo DB Abreu AP Reis AC Montenegro LR Dauber A Beneduzzi D. Central precocious puberty that appears to be sporadic caused by paternally inherited mutations in the imprinted gene makorin ring finger 3. J Clin Endocrinol Metab. (2014) 99:E1097–103. 10.1210/jc.2013-3126 24628548 PMC4037732

[18] Perry J Day F Elks CE Sulem P Thompson Ferreira T. Parent-of-origin-specific allelic associations among 106 genomic loci for age at menarche. Nature. (2014) 514:92–7. 10.1038/nature13545 25231870 PMC4185210

[19] Berg T Silveira MA Moenter SM. Prepubertal development of GABAergic transmission to gonadotropin-releasing hormone (GnRH) neurons and postsynaptic response are altered by prenatal androgenization. J Neurosci. (2018) 38:2283–93. 10.1523/JNEUROSCI.2304-17.2018 29374136 PMC5830516

[20] Keen KL Burich AJ Mitsushima D Kasuya E Terasawa E Effects of pulsatile infusion of the GABAA receptor blocker bicuculline on the onset of puberty in female rhesus monkeys. Endocrinology. (1999) 140:5257–66. 10.1210/en.140.11.5257 10537156

[21] Kanasaki H Oride A Mijiddorj T Sukhbaatar U Kyo S. How is GnRH regulated in GnRH-producing neurons? Studies using GT1-7 cells as a GnRH-producing cell model. Gen Comp Endocrinol. (2017) 247:138–42. 10.1016/j.ygcen.2017.01.025 28131616

[22] Leka-Emiri S Chrousos GP Kanaka-Gantenbein C. The mystery of puberty initiation: genetics and epigenetics of idiopathic central precocious puberty (ICPP). J Endocrinol Invest. (2017) 40:789–802. 10.1007/s40618-017-0627-9 28251550

[23] Wood AR Esko T Yang J Vedantam S Pers TH Gustafsson S. Defining the role of common variation in the genomic and biological architecture of adult human height. Nat Genet. (2014) 46:1173–86. 10.1038/ng.3097 25282103 PMC4250049

[24] Howard SR Dunkel L. Delayed puberty - phenotypic diversity, molecular genetic mechanisms and recent discoveries. Endocr Rev. (2019) 40:1285–317. 10.1210/er.2018-00248 31220230 PMC6736054

[25] Teles MG Silveira LF Tusset C Latronico AC. New genetic factors implicated in human GnRH-dependent precocious puberty: the role of kisspeptin system. Mol Cell Endocrinol. (2011) 346:84–90. 10.1016/j.mce.2011.05.019 21664234

[26] Eaves L Silberg J Foley D Bulik C Maes H Erkanli A. Genetic and environmental influences on the relative timing of pubertal change. Twin Res. (2004) 7:471–81. 10.1375/1369052042335278 15527663

[27] Day FR Thompson DJ Helgason H Chasman DI Finucane H. Genomic analyses identify hundreds of variants associated with age at menarche and support a role for puberty timing in cancer risk. Nat Genet. (2017) 49: 834–41. 10.1038/ng.3841 28436984 PMC5841952

[28] Sørensen K Aksglaede L Petersen JH Juul A. Recent changes in pubertal timing in healthy Danish boys: associations with body mass index. J Clin Endocrinol Metab. (2010) 95:263–70. 10.1210/jc.2009-1478 19926714

[29] Ropelato MG Escobar ME Gottlieb S Bergadá C. Gonadotropin secretion in prepubertal normal and agonadal children evaluated by ultrasensitive time-resolved immunofluorometric assays. Horm Res. (1997) 48:164–72. 10.1159/000185508 9378462

[30] Stamou MI Cox KH Crowley WF Jr. Discovering genes essential to the hypothalamic regulation of human reproduction using a human disease model: adjusting to life in the “-omics” era. Endocr Rev. (2015) 36:603–21. 10.1210/er.2015-1045 26394276 PMC4702497

[31] Day F Perry JRB Ong KK. Genetic regulation of puberty timing in humans Neuroendocrinology. (2015) 102:247–55. 10.1159/000431023 25968239 PMC6309186

[32] Aylwin CF Toro CA Shirtcliff E Lomniczi A. Emerging genetic and epigenetic mechanisms underlying pubertal maturation in adolescence. J Res Adolesc. (2019) 29:54–79. 10.1111/jora.12385 30869843

[33] Bessa DS Maschietto M Aylwin CF Canton APM Brito VN Macedo DB. Methylome profiling of healthy and central precocious puberty girls. Clin Epigenetics. (2018) 10:146. 10.1186/s13148-018-0581-1 30466473 PMC6251202

[34] Kurian JR Keen KL Terasawa E. Epigenetic changes coincide with *in vitro* primate GnRH neuronal maturation. Endocrinology. (2010) 151:5359–68. 10.1210/en.2010-0555 20861233 PMC2954729

[35] Lomniczi A Loche A Castellano JM Ronnekleiv OK Bosch M Kaidar G. Epigenetic control of female puberty. Nat Neurosci. (2013) 16:281–9. 10.1038/nn.3319 23354331 PMC3581714

[36] Yuan X Li Z Ye S Chen Z Huang S Zhong Y. Genome-wide DNA methylation analysis of pituitaries during the initiation of puberty in gilts. PLoS ONE. (2019) 14:e0212630. 10.1371/journal.pone.0212630 30845225 PMC6405085

[37] Lomniczi A Wright H Ojeda SR. Epigenetic regulation of female puberty. Front Neuroendocrinol. (2015) 36:90–107. 10.1016/j.yfrne.2014.08.003 25171849 PMC6824271

[38] Greenspan LC Lee MM. Endocrine disrupters and pubertal timing. Curr Opin Endocrinol Diabetes Obes. (2018) 25:49–54. 10.1097/MED.0000000000000377 29135489 PMC6009831

[39] Leonardi A Cofini M Rigante D Lucchetti L Cipolla C Penta L. The effect of bisphenol A on puberty: a critical review of the medical literature. Int J Environ Res Public Health. (2017) 14:E1044. 10.3390/ijerph14091044 28891963 PMC5615581

[40] Parent AS Franssen D Fudvoye J Pinson A Bourguignon JP. Current changes in pubertal timing: revised vision in relation with environmental factors including endocrine disruptors. Endocr Dev. (2016) 29:174–84. 10.1159/000438885 26680578

[41] Bourguignon JP Juul A Franssen D Fudvoye J Pinson A Parent AS. Contribution of the endocrine perspective in the evaluation of endocrine disrupting chemical effects: the case study of pubertal timing. Horm Res Paediatr. (2016) 86:221–32. 10.1159/000442748 26799415

[42] Euling SY Herman-Giddens ME Lee PA Selevan SG Juul A Sorensen TI. Examination of US puberty-timing data from 1940 to 1994 for secular trends: panel findings. Pediatrics. (2008) 121:S172–91. 10.1542/peds.2007-1813D 18245511

[43] Brix N Ernst A Lauridsen LLB Parner E Støvring H Olsen J. Timing of puberty in boys and girls: a population-based study. Paediatr Perinat Epidemiol. (2019) 33:70–8. 10.1111/ppe.12507 30307620 PMC6378593

[44] Bourguignon JP Franssen D Gérard A Janssen S Pinson A Naveau E. Early neuroendocrine disruption in hypothalamus and hippocampus: developmental effects including female sexual maturation and implications for endocrine disrupting chemical screening. J Neuroendocrinol. (2013) 25:1079–87. 10.1111/jne.12107 24028442

[45] Maranghi F Mantovani A. Targeted toxicological testing to investigate the role of endocrine disrupters in puberty disorders. Reprod Toxicol. (2012) 33:290–6. 10.1016/j.reprotox.2012.01.009 22342511

[46] Li W Liu Q Deng X Chen Y Liu S Story M. Association between obesity and puberty timing: a systematic review and meta-analysis. Int J Environ Res Public Health. (2017) 14:E1266. 10.3390/ijerph14101266 29064384 PMC5664767

[47] Cousminer DL Berry DJ Timpson NJ Ang W Thiering E Byrne EM. Genome-wide association and longitudinal analyses reveal genetic loci linking pubertal height growth, pubertal timing and childhood adiposity. Hum Mol Genet. (2013)22:2735–47. 10.1093/hmg/ddt104 23449627 PMC3674797

[48] Kaplowitz PB. Link between body fat and the timing of puberty. Pediatrics. (2008) 121 (Suppl. 3):S208–17. 10.1542/peds.2007-1813F 18245513

[49] Manfredi-Lozano M Roa J Ruiz-Pino F Piet R Garcia-Galiano D Pineda R. Defining a novel leptin-melanocortin-kisspeptin pathway involved in the metabolic control of puberty. Mol Metab. (2016) 5:844–57. 10.1016/j.molmet.2016.08.003 27688998 PMC5034608

[50] Polyzos SA Mantzoros CS. Leptin in health and disease: facts and expectations at its twentieth anniversary. Metabolism. (2015) 64:5–12. 10.1016/j.metabol.2014.10.017 25467841

[51] Gomes LG Cunha-Silva M Crespo RP Ramos CO Montenegro LR Canton A. DLK1 is a novel link between reproduction and metabolism. J Clin Endocrinol Metab. (2019) 104:2112–20. 10.1210/jc.2018-02010 30462238

[52] Dauber A Cunha-Silva M Macedo DB Brito VN Abreu AP Roberts SA. Paternally inherited DLK1 deletion associated with familial central precocious puberty. J Clin Endocrinol Metab. (2017) 102:1557–67. 10.1210/jc.2016-3677 28324015 PMC5443333

[53] Kundu P Blacher E Elinav E Pettersson S Our gut microbiome: the evolving inner self cell. (2017) 171:1481–93. 10.1016/j.cell.2017.11.024 29245010

[54] McVey Neufeld KA Luczynski P Dinan TG Cryan JF. Reframing the teenage wasteland: adolescent microbiota-gut-brain axis. Can J Psychiatry. (2016) 61:214–21. 10.1177/0706743716635536 27254413 PMC4794958

[55] Cheng HL Sainsbury A Garden F Sritharan M Paxton K Luscombe G. Ghrelin and peptide YY change during puberty: relationships with adolescent growth, development, and obesity. J Clin Endocrinol Metab. (2018) 103:2851–60. 10.1210/jc.2017-01825 29860506

[56] Tena-Sempere M. Ghrelin, the gonadal axis and the onset of puberty. Endocr Dev. (2013) 25:69–82. 10.1159/000346055 23652393

[57] Kharitonenkov A Shiyanova TL Koester A Ford AM Micanovic R Galbreath EJ. FGF-21 as a novel metabolic regulator. J Clin Invest. (2005) 115:1627–35. 10.1172/JCI23606 15902306 PMC1088017

[58] Owen BM Bookout AL Ding X Lin VY Atkin SD Gautron L. FGF21 contributes to neuroendocrine control of female reproduction. Nat Med. (2013) 19:1153–6. 10.1038/nm.3250 23933983 PMC3769455

[59] Livadas S Dracopoulou M Vasileiadi K Lazaropoulou C Magiakou MA Xekouki P. Elevated coagulation and inflammatory markers in adolescents with a history of premature adrenarche. Metabolism. (2009) 58:576–81. 10.1016/j.metabol.2008.12.002 19303981

[60] Utriainen P Laakso S Liimatta J Jaaskelainen J Voutilainen R. Premature adrenarche–a common condition with variable presentation. Horm Res Paediatr. (2015) 83:221–31. 10.1159/000369458 25676474

[61] Katugampola H King PJ Chatterjee S Meso M Duncan AJ Achermann JC. Kisspeptin is a novel regulator of human fetal adrenocortical development and function: a finding with important implications for the human fetoplacental unit. J Clin Endocrinol Metab. (2017) 102:3349–59. 10.1210/jc.2017-00763 28911133 PMC5587078

[62] Voutilainen R Jääskeläinen J. Premature adrenarche: etiology, clinical findings, and consequences. J Steroid Biochem Mol Biol. (2015) 145:226–36. 10.1016/j.jsbmb.2014.06.004 24923732

[63] Ibanez L Oberfield SE Witchel S Auchus RJ Chang RJ Codner E. An international consortium update: pathophysiology, diagnosis and treatment of polycystic ovarian syndrome in adolescence. Horm Res Paediatr. (2017) 88:371–95. 10.1159/000479371 29156452

[64] Jazwiec PA Sloboda DM. Nutritional adversity, sex and reproduction: 30 years of DOHaD and what have we learned? J Endocrinol. (2019) 242:T51–68. 10.1530/JOE-19-0048 31013473

[65] Ellis BJ Del Giudice M. Developmental adaptation to stress: an evolutionary perspective. Annu Rev Psychol. (2019) 70:111–39. 10.1146/annurev-psych-122216-011732 30125133

[66] Iwasa T Matsuzaki T Murakami M Fujisawa S Kinouchi R Gereltsetseg G. Effects of intrauterine undernutrition on hypothalamic Kiss1 expression and the timing of puberty in female rats. J Physiol. (2010) 588:821–9. 10.1113/jphysiol.2009.183558 20083512 PMC2834941

[67] Brix N Ernst A Lauridsen LLB Arah OA Nohr EA Olsen J Maternal pre-pregnancy obesity and timing of puberty in sons and daughters: a population-based cohort study. Int J Epidemiol. (2019) 25:dyz125 10.1093/ije/dyz125 PMC6857762 31237934

[68] Villamor E Jansen EC. Nutritional determinants of the timing of puberty. Annu Rev Public Health. (2016) 37:33–46. 10.1146/annurev-publhealth-031914-122606 26789387

[69] Neville KJ Walker J. Precocious pubarche is associated with SGA, prematurity, weight gain, and obesity. Arch Dis Child. (2005) 90: 258–61. 10.1136/adc.2004.053959 15723910 PMC1720316

[70] Ong K de Zegher F Valls C Dunger DB Ibáñez L. Persisting benefits 12–18 months after. discontinuation of pubertal metformin therapy in low birthweight girls. Clin Endocrinol. (2007) 67:468–71. 10.1111/j.1365-2265.2007.02952.x 17608755 PMC2040227

[71] Ibanez L de Zegher F Potau N. Anovulation after precocious pubarche: early markers and time course in adolescence. J Clin Endocrinol Metab. (1999) 84:2691–5. 10.1210/jc.84.8.2691 10443661

[72] Virdis R Street M Zampolli M Radetti G Pezzini B Benelli M. Precocious puberty in girls adopted from developing countries. Arch Dis Child. (1998) 78:152–4. 10.1136/adc.78.2.152 9579158 PMC1717454

[73] Yackobovitch-Gavan M Fisch Shvalb N Bhutta ZA. Malnutrition and catch-up growth during childhood and puberty. World Rev Nutr Diet. (2018) 117:129–50. 10.1159/000484503 29393117

[74] Gur RE Moore TM Rosen AFG Barzilay R Roalf DR Calkins ME. Burden of environmental adversity associated with psychopathology, maturation, and brain behavior parameters in youths. JAMA Psychiatry. (2019). 10.1001/jamapsychiatry.2019.0943. [Epub ahead of print]. 31141099 PMC6547104

[75] Colich NL Platt JM Keyes KM Sumner JA Allen NB McLaughlin KA Earlier age at menarche as a transdiagnostic mechanism linking childhood trauma with multiple forms of psychopathology in adolescent girls. Psychol Med. (2019) 25:1–9. 10.1017/S0033291719000953 PMC6814488 31020943

[76] Callaghan BL Tottenham N. The stress acceleration hypothesis: effects of early-life adversity on emotion circuits and behavior. Curr Opin Behav Sci. (2016) 7:76–81. 10.1016/j.cobeha.2015.11.018 29644262 PMC5890821

[77] Steinberg GR. Cellular energy sensing and metabolism-implications for treating diabetes: the 2017 outstanding scientific achievement award lecture. Diabetes. (2018) 67:169–79. 10.2337/dbi17-0039 29358486

[78] Sabatini DM. Twenty-five years of mTOR: uncovering the link from nutrients to growth. PNAS. (2017) 114:11818–25. 10.1073/pnas.1716173114 29078414 PMC5692607

[79] Saxton RA Sabatini DM. mTOR signaling in growth, metabolism, and disease. Cell. (2017) 168:960–76. 10.1016/j.cell.2017.02.004 28283069 PMC5394987

[80] Lu M Sarruf DA Li P Osborn O Sanchez-Alavez M Talukdar S. Neuronal Sirt1 deficiency increases insulin sensitivity in both brain and peripheral tissues. J Biol Chem. (2013) 288:10722–35. 10.1074/jbc.M112.443606 23457303 PMC3624452

[81] Toorie AM Nillni EA. Minireview: central Sirt1 regulates energy balance via the melanocortin system and alternate pathways. Mol Endocrinol. (2014) 28:1423–34. 10.1210/me.2014-1115 24947673 PMC4154241

[82] Vazquez MJ Toro CA Castellano JM Ruiz-Pino F Roa J Beiroa D. SIRT1 mediates obesity- and nutrient-dependent perturbation of pubertal timing by epigenetically controlling Kiss1 expression. Nat Commun. (2018) 9:4194. 10.1038/s41467-018-06459-9 30305620 PMC6179991

[83] Choi I Rickert E Fernandez M Webster NJG. SIRT1 in astrocytes regulates glucose metabolism and reproductive function. Endocrinology. (2019) 160:1547–60. 10.1210/en.2019-00223 31127273 PMC6542483

[84] Heindel JJ Blumberg B Cave M Machtinger R Mantovani A Mendez MM. Metabolism disrupting chemicals and metabolic disorders. Reprod Toxicol. (2017) 68:3–33. 10.1016/j.reprotox.2016.10.001 27760374 PMC5365353

[85] Marraudino M Bonaldo B Farinetti A Panzica G Ponti G Gotti S. Metabolism disrupting chemicals and alteration of neuroendocrine circuits controlling food intake and energy metabolism. Front Endocrinol. (2019) 9:766. 10.3389/fendo.2018.00766 30687229 PMC6333703

[86] Xue J Ideraabdullah FY. An assessment of molecular pathways of obesity susceptible to nutrient, toxicant and genetically induced epigenetic perturbation. J Nutr Biochem. (2016) 30:1–13. 10.1016/j.jnutbio.2015.09.002 27012616 PMC4808242

[87] Fishman SL Sonmez H Basman C Singh V Poretsky L. The role of advanced glycation end-products in the development of coronary artery disease in patients with and without diabetes mellitus: a review. Mol Med. (2018) 24:59. 10.1186/s10020-018-0060-3 30470170 PMC6251169

[88] Ott C Jacobs K Haucke E Santos AN Grune T Simm A. Role of advanced glycation end products in cellular signaling. Redox Biol. (2014) 2:411–29. 10.1016/j.redox.2013.12.016 24624331 PMC3949097

[89] Brehm E Flaws JA. Transgenerational effects of endocrine-disrupting chemicals on male and female reproduction. Endocrinology. (2019) 160:1421–35. 10.1210/en.2019-00034 30998239 PMC6525581

[90] Vazquez MJ Velasco I Tena-Sempere M. Novel mechanisms for the metabolic control of puberty: implications for pubertal alterations in early-onset obesity and malnutrition. J Endocrinol. (2019) JOE-19-0223.R1. 10.1530/JOE-19-0223. [Epub ahead of print]. 31189134

[91] Sloboda DM Hickey M Hart R. Reproduction in females: the role of the early life environment. Hum Reprod Update. (2011) 17:210–27. 10.1093/humupd/dmq048 20961922

[92] Campbell B. Adrenarche in comparative perspective. Am J Hum Biol. (2011) 23:44–52. 10.1002/ajhb.21111 21140467

[93] Kim C Harrall KK Glueck DH Shumer D Dabelea D Childhood adiposity and adolescent sex steroids in the EPOCH (Exploring perinatal outcomes among children) study. Clin Endocrinol. (2019) 91:525–33. 10.1111/cen.14058 PMC6744341 31278867

[94] Castellano JM Bentsen AH Sánchez-Garrido MA Ruiz-Pino F Romero M Garcia-Galiano D. Early metabolic programming of puberty onset: impact of changes in postnatal feeding and rearing conditions on the timing of puberty and development of the hypothalamic kisspeptin system. Endocrinology. (2011) 152:3396–408. 10.1210/en.2010-1415 21712362

[95] Díaz M Bassols J López-Bermejo A de Zegher F Ibáñez L. Metformin treatment to reduce central adiposity after prenatal growth restraint: a placebo-controlled pilot study in prepubertal children. Pediatr Diabetes. (2015) 16:538–45. 10.1111/pedi.12220 25332100

[96] Sasaki T. Age-associated weight gain, leptin, and SIRT1: a possible role for hypothalamic SIRT1 in the prevention of weight gain and aging through modulation of leptin sensitivity. Front Endocrinol. (2015) 6:109. 10.3389/fendo.2015.00109 26236282 PMC4504171

[97] Nakahata Y Kaluzova M Grimaldi B Sahar S Hirayama J Chen D. “The NAD+-dependent deacetylase SIRT1 modulates CLOCK-mediated chromatin remodeling and circadian control”. Cell. (2008) 134: 329–40. 10.1016/j.cell.2008.07.002 18662547 PMC3526943

[98] Godfrey KM Sheppard A Gluckman PD Lillycrop KA Burdge GC McLean C. Epigenetic gene promoter methylation at birth is associated with child's later adiposity. Diabetes. (2011) 60:1528–34. 10.2337/db10-0979 21471513 PMC3115550

[99] Darnaudéry M Maccari S. Epigenetic programming of the stress response in male and female rats by prenatal restraint stress. Brain Res Rev. (2008) 57:571–85. 10.1016/j.brainresrev.2007.11.004 18164765

[100] Slater-Jefferies JL Lillycrop KA Townsend PA Torrens C Hoile SP Hanson MA. Feeding a protein-restricted diet during pregnancy induces altered epigenetic regulation of peroxisomal proliferator-activated receptor-α in the heart of the offspring. J Dev Orig Health Dis. (2011) 2:250–5. 10.1017/S2040174410000425 22003431 PMC3191520

[101] Day FR Elks CE Murray A Ong KK Perry JR. Puberty timing associated with diabetes, cardiovascular disease and also diverse health outcomes in men and women: the UK Biobank study. Sci Rep. (2015) 5:11208. 10.1038/srep11208 26084728 PMC4471670

